# Social determinants and lifestyle risk factors only partially explain the higher prevalence of food insecurity among Aboriginal and Torres Strait Islanders in the Australian state of Victoria: a cross-sectional study

**DOI:** 10.1186/1471-2458-14-598

**Published:** 2014-06-12

**Authors:** Alison Markwick, Zahid Ansari, Mary Sullivan, John McNeil

**Affiliations:** 1 Department of Health, Health Intelligence Unit, Prevention and Population Health Branch, 50 Lonsdale Street, Melbourne, Victoria 3000, Australia; 2 Department of Health, Aboriginal Health Branch, 50 Lonsdale Street, Melbourne, Victoria 3000, Australia; 3 Department of Epidemiology and Preventive Medicine, School of Public Health and Preventive Medicine, Monash University, 99 Commercial Rd, Melbourne, Victoria 3004, Australia

## Abstract

**Background:**

The prevalence of food insecurity is substantially higher among Australians of Aboriginal or Torres Strait Islander descent. The purpose of this study is to explain the relationship between food insecurity and Aboriginal and Torres Islander status in the state of Victoria.

**Methods:**

Data were obtained from the 2008 Victorian Population Health Survey; a cross-sectional landline computer-assisted telephone interview survey of 34,168 randomly selected Victorians aged 18 years and older; including 339 Aboriginal and Torres Strait Islanders. We categorised a respondent as food insecure, if in the previous 12 months, they reported having run out of food and not being able to afford to buy more. We used multivariable logistic regression to adjust for age, sex, socioeconomic status (household income), lifestyle risk factors (smoking, alcohol consumption and obesity), social support (ability to get help from family, friends or neighbours), household composition (lone parent status, household with a child, and household size), and geographic location (rurality).

**Results:**

Aboriginal and Torres Strait Islanders (20.3%) were more likely than their non-Aboriginal and Torres Strait Islander counterparts (5.4%) to have experienced food insecurity; odds ratio (OR) = 4.5 (95% CI; 2.7-7.4). Controlling for age, SES, smoking, obesity and inability to get help from family or friends reduced the odds ratio by 38%; OR_adjusted_ = 2.8 (1.6-5.0).

**Conclusions:**

Social determinants and lifestyle risk factors only partially explained the higher prevalence of food insecurity among Aboriginal and Torres Strait Islanders in Victoria. Further research is needed to explain the disparity in food insecurity between the two populations in order to inform and guide corrective action.

## Background

Food insecurity has been defined as the *“limited or uncertain availability of nutritionally adequate and safe foods or limited or uncertain ability to acquire acceptable foods in socially acceptable ways”*[1].

The prevalence of food insecurity in developed countries ranges from 4 to 14% [2]. In Australia, a single item question; “In the last 12 months, were there any times that you ran out of food and couldn’t afford to buy more?” was first included in the 1995 *National Nutrition Survey* to estimate the prevalence of food insecurity [2]. Subsequently the question was incorporated into the National Health Survey program and the prevalence of food insecurity in Australia was reported to be approximately 5%; the unemployed, low income households, lone parents and young people being over-represented [3].

The *National Aboriginal and Torres Strait Islander Health Survey* in 2004–05 found that the prevalence of food insecurity was higher (24%) among Australians of Aboriginal and Torres Strait Islander descent [4]. In the second most populous Australian state of Victoria, the substantially higher prevalence of food insecurity among Aboriginal and Torres Strait Islanders, adjusted for age, was found to be 17.7% (crude prevalence = 20.3%) [5].

It is well established that Aboriginal and Torres Strait Islander Australians are notably more socially and economically disadvantaged than their non-Aboriginal and Torres Strait Islander counterparts [6]. There is some evidence, however, that the main cause of food insecurity among Aboriginal and Torres Strait Islanders who live in remote rural communities is food scarcity rather than economic constraint [7]. However, the state of Victoria does not have any remote rural communities. Therefore, we hypothesised that the substantial difference in prevalence of food insecurity between Aboriginal and Torres Strait Islanders and their non-Aboriginal and Torres Strait Islander counterparts in Victoria would primarily be explained by economic constraint [8].

The aim of this paper was to investigate the relationship between food insecurity and Aboriginal and Torres Strait Islander status in the state of Victoria. More specifically, this paper aims to: (1) identify factors associated with food insecurity in Victoria; (2) measure the strength of the association between Aboriginal and Torres Strait Islander status and food insecurity; and (3) describe the influence of other factors associated with food insecurity on the association between Aboriginal and Torres Strait Islander status and food insecurity.

## Methods

### Data source, sampling frame and procedures

Data were collected as part of the Victorian Population Health Survey (VPHS) in 2008; a cross-sectional state-wide survey conducted to provide information on the health and well-being of Victorians [5]. The survey was conducted by computer-assisted telephone interview, on a randomly selected representative sample of people aged 18 years and older, who resided in private dwellings in Victoria and had access to a landline telephone. The sampling frame was an electronic listing of Victorian telephone exchange prefixes and localities and random digit dialling was used to generate a sample of telephone numbers that formed the household sample. Only one person aged 18 years and older per household was selected for interview and that was the person with the most recent birthday.

### Sample size

The survey sample was stratified by Local Government Area (LGA), of which there are 79 LGAs in the state of Victoria, with a target sample of 426 interviews per LGA. The total sample achieved was 34,168 completed interviews, including 339 respondents of Aboriginal and/or Torres Strait Islander descent. The sample size was based on the following formula assuming a prevalence of 7.5 per cent for a variable of interest, with a confidence interval of 2.5 per cent [i.e. 7.5 (5.0, 10.0) per cent]:

Sample size (n) = Z^2^ * p * (1 – p)/c^2^ = 426, where p = proportion (0.075), Z = 1.96 [Z-score of level of significance (alpha = 0.05)], c = confidence interval (0.025) [9].

### Response rate

The response rate, defined as the proportion of households where contact was made and an interview completed, was 64.9%.

### Ethics statement

The Department of Health Human Research Ethics Committee approved the survey in accordance with the guidelines of the Declaration of Helsinki.

### Variables

Food insecurity status was the outcome variable. Independent variables that were hypothesized a priori to be associated with food insecurity included; age, sex, socioeconomic status (total annual household income), lifestyle risk factors (smoking, excessive alcohol consumption and obesity), social support (inability to get help from family, neighbours or friends), household composition (lone parenthood, household with a child and household size), and geographic location (rurality).

A respondent was judged to be food insecure if they responded in the affirmative to the question: “In the last 12 months, were there any times that you ran out of food, and couldn’t afford to buy more?”

Aboriginal and Torres Strait Islander status was determined by asking the question “Are you of Aboriginal or Torres Strait Islander origin?” Respondents who stated that they were Aboriginal (n = 258), Torres Strait Islander (n = 40) or both (n = 41) were combined and a binary variable created.

Socioeconomic status (SES) was measured by total annual household income which included income before tax from all sources such as social security payments, child support, and investments over the previous 12 months.

Survey respondents were asked about the lifestyle risk factors of smoking, excessive alcohol consumption and obesity. Excessive alcohol consumption was determined by comparing the respondent’s pattern of alcohol consumption with the 2001 Australian recommended guidelines [10]. Those that exceeded the level of consumption considered to be safe were considered to be risky drinkers. Obesity was defined as a body mass index (BMI) of 30 kg/m^2^ or greater according to the recommendations of the World Health Organization; calculated from self-reported height and weight.

Social support was assessed by asking the respondent whether they could get help from family, neighbours or friends when needed.

Rurality was defined as being resident outside the metropolitan area of Melbourne within the state of Victoria.

### Coding of variables

All independent variables, with the exception of household income, age and household size, were binary and the code 0 represented the referent group. Aboriginal and Torres Strait Islander status, smoking, excessive alcohol consumption, obesity, lone parenthood and households with a child were coded as 0 = no, 1 = yes. Sex was coded as 0 = male, 1 = female. The ability to get help from family, friends or neighbours was coded as 0 = yes, 1 = no. Rurality was coded as 0 = metropolitan, 1 = rural. Income and age were categorical with the highest income and oldest age category as the referent groups. Household size was a continuous variable.

### Missing data

Less than 1% of respondents refused to answer or were unable to answer the survey questions for all variables; with the exception of total annual household income (18%), self-reported height and weight (6%) and the ability to get help from neighbours (3%). Missing data was excluded from the analysis. The models were rerun with the missing data included, but this made negligible difference to the findings.

### Weighting

In order to control for participation bias, the survey data were weighted to reflect the age/sex/geographic distribution of the estimated resident population of Victoria and the probability of selection of the household and respondent within the household. The data were not weighted for ethnicity as this was not part of the original study design, which was to provide prevalence estimates for the whole population by LGA.

### Statistical analysis

We analysed the survey data using the Stata statistical software package version 12 [11]. Initially we used univariable logistic regression to identify factors that were associated with food insecurity. Then we used multivariable logistic regression to investigate the relationship between food insecurity and Aboriginal and Torres Strait Islander status adjusting for all other factors that were associated with food insecurity. The outcome variable was whether or not a person reported at least one episode of food insecurity in the previous 12 months. The independent variable of interest was Aboriginal and Torres Strait Islander status with non-Aboriginal and Torres Strait Islanders as the referent group. Statistical significance was determined at the p < 0.05 level. Statistical reliability was assessed by calculating relative standard errors (RSEs) for all prevalence estimates and RSEs of less than 25% were deemed to indicate an acceptable level of reliability. Variables that were not statistically significant in the univariable logistic regression analyses were not included in the multivariable logistic regression analyses. We tested for interaction by fitting interaction terms between the main variable of interest (Aboriginal or Torres Strait Islander status) and all factors that were found to be statistically significantly associated with food insecurity. We examined the adequacy of the final model in which all factors that were associated with food insecurity were included, using the Hosmer-Lemeshow goodness-of-fit test developed specifically for complex survey data [12].

## Results

Table 1 shows the demographic characteristics of the Victorian population by Aboriginal and Torres Strait Islander status. Overall, the Aboriginal and Torres Strait Islander population was younger with a greater proportion residing in rural Victoria, compared with the non-Aboriginal and Torres Strait Islander population.

**Table 1:** Demographic characteristics of Victoria, by Aboriginal and Torres Strait Islander status

**Demographic characteristic**	ATSI *		Non-ATSI *	
	n **	**Weighted% (95% CI)**	n **	**Weighted% (95% CI)**
Males	120	51.4 (42.6–60.1)	12,830	48.9 (48.0–49.8)
Females	219	48.6 (39.9–57.4)	20,909	51.1 (50.2–52.0)
18-34 years	84	43.5 (34.6–52.9)	4,710	31.2 (30.3–32.1)
35-64 years	195	45.1 (36.6–53.9)	19,550	51.3 (50.4–52.2)
65 years and older	60	11.3 (7.7–16.4)	9,479	17.5 (17.0–18.0)
Resident in metropolitan Victoria	96	64.8 (57.0–71.9)	13,474	73.8 (73.4–74.2)
Resident in rural Victoria	243	35.2 (28.1–43.0)	20,265	26.2 (25.8–26.6)

ATSI * = Aboriginal and Torres Strait Islander.

**Unweighted sample.

95% CI = 95 per cent confidence interval.

Metropolitan Victoria consists of the City of Melbourne and its environs.

The crude prevalence of food insecurity in Victoria (Table 2) was substantially higher among Aboriginal and Torres Strait Islanders (20.3%), compared with their non-Aboriginal and Torres Strait Islander counterparts (5.4%).

**Table 2:** Prevalence of social determinants and lifestyle risk factors by food insecurity status

**Independent variable**	**Food secure**		**Food insecure**		**Univariable analysis**	
	**N**^**b**^	**Weighted% (95% CI)**	**N**^**b**^	**Weighted% (95% CI)**	**Crude OR (95% CI)**	**p value**
***ATSI***^***a ***^ ***status***						
No	31,820	94.6 (94.2-95.0)	1,871	5.4 (5.0-5.8)	1.00	
Yes	280	82.3 (75.2-87.7)	59	20.3 (13.5-29.4)	4.5 (2.7-7.4)	<0.001
***Age***^***c ***^ ***and sex***						
65+ years	9,355	97.7 (97.2-98.1)	201	2.3 (1.9-2.8)	1.00	
55-64 years	6,967	97.2 (96.7-97.6)	312	2.8 (2.4-3.3)	1.2 (0.9-1.6)	0.15
45-54 years	6,190	94.6 (93.7-95.3)	481	5.4 (4.7-6.3)	2.4 (1.9-3.1)	<0.001
35-44 years	5,320	93.3 (92.4-94.1)	485	6.7 (5.9-7.6)	3.0 (2.4-3.9)	<0.001
25-34 years	2,902	91.9 (90.4-93.2)	314	8.1 (6.8-9.6)	3.7 (2.8-4.9)	<0.001
18-24 years	1,436	92.5 (90.7-94.0)	149	7.5 (6.0-9.3)	3.4 (2.5-4.6)	<0.001
Male	12,416	95.4 (94.7-96.0)	551	4.6 (4.0-5.3)	1.00	
Female	19,754	93.6 (93.0-94.1)	1,391	6.4 (5.9-7.0)	1.4 (1.2-1.7)	<0.001
***Socioeconomic status***^***d***^						
Greater than $80,000	6,711	98.1 (97.5-98.6)	100	1.9 (1.4-2.5)	1.00	
$60,001 - $80,000	3,692	96.8 (95.8-97.6)	103	3.2 (2.4-4.2)	1.7 (1.1-2.5)	0.01
$40,001 - $60,000	4,646	94.2 (92.9-95.3)	224	5.8 (4.7-7.1)	3.2 (2.2-4.5)	<0.001
$20,001 - $40,000	6,843	90.7 (89.4-92.0)	525	9.3 (8.0-10.6)	5.2 (3.8-7.3)	<0.001
$20,000 or less	5,332	85.9 (84.2-87.5)	776	14.1 (12.5-15.8)	8.4 (6.1-11.6)	<0.001
***Lifestyle risk factors***						
Non or ex-smoker	26,918	96.1 (95.7-96.5)	1,079	3.9 (3.5-4.3)	1.00	
Smoker	5,119	87.6 (86.1-88.9)	856	12.4 (11.1-13.9)	3.5 (3.0-4.1)	<0.001
Risky drinker - no	19,426	94.7 (94.1-95.2)	1,124	5.3 (4.8-5.9)	1.00	
Risky drinker - yes	12,528	94.2 (93.5-94.9)	805	5.8 (5.1-6.5)	1.1 (0.9-1.3)	0.29
Not obese	24,131	95.1 (94.6-95.6)	1,246	4.9 (4.4-5.4)	1.00	
Obese	6,100	92.3 (91.1-93.3)	525	7.7 (6.7-8.9)	1.6 (1.4-2.0)	<0.001
***Social support***^***e***^						
Family - yes	29,434	95.2 (94.8-95.6)	1,460	4.8 (4.4-5.2)	1.00	
Family - no	2,598	85.2 (82.8-87.4)	466	14.8 (12.6-17.2)	3.5 (2.8-4.3)	<0.001
Neighbours - yes	25,301	96.0 (95.5-96.3)	1,442	4.0 (3.7-4.5)	1.00	
Neighbours - no	5,899	90.4 (89.2-91.5)	731	9.6 (8.5-10.8)	2.5 (2.1-3.0)	<0.001
Friends - yes	30,522	95.0 (94.6-95.4)	1,625	5.0 (4.6-5.4)	1.00	
Friends - no	1,424	84.0 (81.3-86.4)	302	16.0 (13.6-18.7)	3.6 (3.0-4.5)	<0.001
***Household composition***						
Not a lone parent	30,618	95.0 (94.6-95.4)	1,581	5.0 (4.6-5.4)	1.00	
Lone parent	1,431	82.5 (79.7-84.9)	350	17.5 (15.1-20.3)	4.0 (3.3-4.9)	<0.001
Childless household	22,229	95.4 (94.8-95.8)	1,045	4.6 (4.2-5.2)	1.00	
Household with a child	9,851	93.1 (92.4-93.8)	890	6.9 (6.2-7.6)	1.5 (1.3-1.8)	<0.001
Household size^f^	32,170		1,942		1.1 (1.1-1.2)	<0.001
***Geographic location***						
Metropolitan	12,842	94.5 (93.9-95.0)	743	5.5 (5.0-6.1)	1.00	
Rural	19,328	94.5 (93.9-95.0)	1,199	5.5 (5.0-6.1)	1.0 (0.9-1.2)	0.99

95% CI = 95 per cent confidence interval, OR = odds ratio.

^a^ATSI = Aboriginal and Torres Strait Islander.

^b^N = raw unweighted sample size; however, prevalence and prevalence odds ratio estimates are based on weighted data.

^c^Mean age = 45.7 years (45.6-45.8); min = 18 years, max = 99.

^d^Total annual household income.

^e^Ability to get help from family, neighbours or friends, when needed.

^f^Continuous variable.

Table 2 shows the results of the univariable logistic regression analysis that sought to identify factors that were associated with food insecurity. Excessive alcohol consumption and geographic location (rurality) were not associated with food insecurity. The remaining variables all showed associations with food insecurity.

Adult Victorians of Aboriginal and Torres Strait Islander descent were more than four times more likely to have experienced at least one episode of food insecurity compared with their non-Aboriginal and Torres Strait Islander counterparts, odds ratio (OR) = 4.5(95% confidence interval (CI); 2.7-7.4).

Food insecurity was more likely to be experienced by females than males (OR = 1.4; 1.2-1.7). Age was associated with food insecurity with persons aged 18–24 years (OR = 3.4; 2.5-4.6), 25–34 years (OR = 3.7; 2.8-4.9), 35–44 years (OR = 3.0; 2.4-3.9), and 45–54 years (OR = 2.4; 1.9-3.1) being more likely to have experienced food insecurity compared with those aged 65 years and older, while those aged 55–64 years were no different to those aged 65 years and older (OR = 1.2; 0.9-1.6).

SES, measured by total annual household income, was strongly associated with food insecurity. For every decrease in income bracket, food insecurity increased by 70% (OR_trend_ = 1.7; 1.6-1.8).

Of the lifestyle risk factors evaluated, only smoking (OR = 3.5; 3.0-4.1) and obesity (OR = 1.6; 1.4-2.0) were associated with food insecurity.

All three indicators of social support (inability to get help from family or neighbours or friends) were associated with food insecurity; friends (OR = 3.6; 3.0-4.4), family (OR = 3.5; 2.8-4.3) and neighbours (OR = 2.5; 2.1-3.0).

All three indicators of household composition were associated with food insecurity; being a lone parent (OR = 4.0; 3.3-4.9), households with dependent children (OR = 1.5; 1.3-1.8), and household size (OR = 1.1; 1.1-1.2).

Table 3 summarises the relationship of food insecurity to Aboriginal and Torres Strait Islander status after adjustment for other factors associated with food insecurity.

**Table 3:** Relationship between food insecurity and Aboriginal or Torres Strait Islander status: adjusted for social determinants and lifestyle risk factors

**Secondary independent variables**	**Adjusted Odds Ratio (95% CI)**		
	Non-ATSI *	ATSI *	**p value**
***Age and sex***			
Age	1.0	4.0 (2.5-6.6)	<0.001
Sex	1.0	4.6 (2.8-7.5)	<0.001
***Socioeconomic status (SES)***^***a***^	1.0	3.9 (2.3-6.6)	<0.001
***Lifestyle risk factors***^***b***^			
Current smoker	1.0	3.8 (2.3-6.3)	<0.001
Obese	1.0	4.9 (2.9-8.2)	<0.001
***Social support***^***c***^			
Unable to get help from family	1.0	4.1 (2.5-6.9)	<0.001
Unable to get help from friends	1.0	4.8 (2.9-7.9)	<0.001
Unable to get help from neighbours	1.0	4.5 (2.7-7.5)	<0.001
***Household composition***^***d***^			
Lone parent household	1.0	4.6 (2.8-7.6)	<0.001
Household with a child	1.0	4.4 (2.7-7.2)	<0.001
Household size	1.0	4.6 (2.8-7.3)	<0.001
**Multivariable models**			
Age and SES^a^	1.0	3.0 (1.8-5.2)	<0.001
Age and lifestyle risk factors^b^	1.0	3.8 (2.3-6.4)	<0.001
Age and social support^c^	1.0	4.0 (2.4-6.6)	<0.001
Age, SES and lifestyle risk factors^c b^	1.0	2.9 (1.6-5.2)	<0.001
Age, SES and social support^a c^	1.0	2.9 (1.7-5.1)	<0.001
Age, SES, lifestyle & social support^a b c^	1.0	2.8 (1.6-5.0)	0.001
All significant predictor variables^d e^	1.0	2.9 (1.6-5.1)	<0.001

ATSI *= Aboriginal and Torres Strait Islander

Crude odds ratio was 4.5 (2.7-7.4).

^a^Total annual household income, ^b^Smoking status and obesity, ^c^Inability to get help from family and/or friends, ^d^All variables shown by univariable analysis to be significantly (p < 0.05) associated with food insecurity.

^e^Hosmer-Lemeshow goodness-of-fit test; F(9, 26118) = 0.55, prob > F = 0.8359. All interaction terms were insignificant. All p values were <0.001 for the adjusted odds ratio.

Controlling for sex had no impact on the crude OR. By contrast, the adjusted OR (OR_adj_) when age was taken into account was 4.0 (2.5-6.6), representing an 11.1% reduction of the crude OR.

Controlling for SES (total annual household income) reduced the crude OR by 13.3% (OR_adj_ = 3.9; 2.3-6.6).

Of the two lifestyle variables, controlling for smoking status reduced the crude OR by 15.6% (OR_adj_ = 3.8; 2.3-6.3). By contrast, controlling for obesity increased the crude OR by 8.9% (OR_adj_ = 4.9; 2.9-8.3).

Of the three social support variables, controlling for the inability to get help from neighbours had no impact on the crude OR. By contrast, controlling for being unable to get help from family reduced the crude OR by 8.9% (OR_adj_ = 4.1; 2.5-6.9) and controlling for being unable to get help from friends increased the crude OR by 6.7% (OR_adj_ = 4.8, 2.9-7.9).

Of the three household composition variables, controlling for being a lone parent household, households with a child or household size had no impact on the crude OR.

Subsequently, we fitted various multivariable models to examine the relative impacts of each of the domains of the factors that were associated with food insecurity. Age and sex, SES and lifestyle risk factors were the only domains that appeared to make a substantial impact as measured by a change in the crude OR. However, when the individual factors were examined, the impact of sex and obesity was small if present at all, while the impact of the inability to get help from friends appeared to cancel the impact on the inability to get help from family because their effects were in opposite directions.

We ran a multivariable model that included only the variables that changed the crude OR by 5% or more: age, smoking status, obesity, inability to get help from family and/or friends. This reduced the association of food insecurity and Aboriginal and Torres Strait Islander status by approximately 38% from OR = 4.5 (2.7-7.4) to OR_adj_ = 2.8 (1.6-5.0). The model provided a good fit to the data, by the *F*-adjusted mean residual goodness-of-fit test: F(9,26972) = 1.28, prob > F = 0.2413. When we included all the other factors that were associated with food insecurity, irrespective of whether they impacted on the crude OR, we obtained similar results.

All first order interaction terms with the primary explanatory variable were not statistically significant and therefore not included in the model.

## Discussion

We investigated the relationship between food insecurity and Aboriginal and Torres Strait Islander status and show that Aboriginal and Torres Strait Islanders in Victoria were markedly more likely to have experienced food insecurity than their non-Aboriginal and Torres Strait Islander counterparts. Age, household income, smoking, obesity, and the inability to get help from family and/or friends only partially explained the higher prevalence of food insecurity among Aboriginal and Torres Strait Islanders.

Remote and rural communities may have problems accessing food due to geographical barriers; resulting in food scarcity, which has been identified as a major cause of food insecurity among Aboriginal and Torres Strait Islander populations in Australia [7]. Our finding that rurality (being resident in rural Victoria) was not associated with food insecurity supports our hypothesis that food scarcity is not a significant determinant of food insecurity in the state of Victoria, and highlights the importance of not assuming that the determinants of health and well-being among Aboriginal and Torres Strait Islanders are the same across the country.

The finding that younger age is associated with food insecurity, while older age appears to be protective is consistent with the literature [8]. This may reflect the fact that Australia provides a modest safety net for the elderly that is often lacking for the younger age groups. Since the Aboriginal and Torres Strait Islander population is a much younger population, it is therefore to be expected that this would render Aboriginal and Torres Strait Islanders more vulnerable to food insecurity.

Total annual household income and smoking made the largest contributions to the relationship between food insecurity and Aboriginal and Torres Strait Islander status; reducing the OR by approximately 13% and 16%, respectively. Anglicare Australia, in their recent study of the clientele at emergency relief centres, concluded that insufficient income is the key determinant of food insecurity in Australia, as food is often the only discretionary item in a low-income household budget [8]. Our findings confirm the importance of income; as the lower the household income the higher the OR of food insecurity, with those in the lowest household income bracket being 8.4 times more likely to have experienced food insecurity compared with those in the highest household income bracket. However, while household income is often used as a proxy of SES, it does not capture all aspects of SES [13]. SES is a multi-dimensional concept and other indicators of SES include: education, occupation, wealth, and area-based composite measures. These indicators are not necessarily interchangeable as they do not always correlate highly with each other; for example, the correlation between income and education has been reported to vary by ethnic group from 0.34 to 0.58, reflecting that income can vary at similar levels of education across different ethnic groups [14]. We selected household income as a measure of SES, based on our consideration of the most plausible causal pathway between the outcome variable of food insecurity and SES; since, income provides individuals and families with the necessary material resources and determines their purchasing power for accessing goods and services. However, it is possible that the difference in prevalence of food insecurity between Aboriginal and Torres Strait Islanders and their non- Aboriginal and Torres Strait Islander counterparts, after adjusting for household income, may still reflect unmeasured socioeconomic differences not captured by household income. Moreover, income as a measure of SES has been shown to be a more unstable measure than education or occupation with a higher non-response rate than other measures [15].

The prevalence of smoking has consistently been reported to be higher in the Aboriginal and Torres Strait Islander population [5]. Hypothetically, the high cost of tobacco products might be expected to put strain on low income households and to be in direct competition with other discretionary household items. However, adjusting for smoking status only reduced the OR by 15.6%; suggesting that smoking did not make a particularly large contribution to the higher prevalence of food insecurity among Aboriginal and Torres Strait Islanders. Given that this is a cross-sectional study design, one cannot rule out the possibility of reverse causation where the stress of being subject to food insecurity causes people to smoke.

Excessive alcohol consumption was not associated and made no difference to our findings whether included or not. The wider societal belief that Aboriginal and Torres Strait Islanders are more likely to engage in excessive alcohol consumption has been the basis of negative stereotyping that has fuelled racist perceptions. This belief, however, is not supported by the evidence. Our data found that the proportion of adult Victorians who engaged in excessive alcohol consumption was no different between Aboriginal and Torres Strait Islanders compared with non-Aboriginal and Torres Strait Islanders (43.9% vs. 45.5%). By contrast, Aboriginal and Torres Strait Islander men were more likely to abstain from alcohol consumption than their non-Aboriginal and Torres Strait Islander counterparts (22.4% vs. 12.4%, p < 0.05) [5]. These findings make an important contribution to dispelling ongoing negative stereotypes and combating racism, given that racism is a key determinant of Aboriginal and Torres Strait Islander health and wellbeing [16].

Aboriginal and Torres Strait Islanders in Victoria bore a higher burden of child removal from their families than any other state in Australia due to previous government assimilation policies [17]. It is possible that our finding that Aboriginal and Torres Strait Islander Victorians were less able to get help from family than their non-Aboriginal and Torres Strait Islander counterparts may be a reflection of the success of such past policies. Since family is usually the first point of contact in times of personal crisis, lacking family support means lacking an important resource that can effectively reduce an individual’s vulnerability to negative life outcomes such as food insecurity. The assimilation policies of previous governments are now recognised as a form of institutional racism and exemplify how racism continues to be a key determinant of Aboriginal and Torres Strait Islander health and well-being, as their impacts continue to be experienced by the current generation [18].

Indigenous people in other comparable countries, such as Canada and New Zealand, have also been reported to experience a higher prevalence of food insecurity than their non-indigenous counterparts [19,20]. A study in Canada showed similar findings to our study; where, adjusting for SES reduced but did not eliminate the difference in prevalence [19]. This study used several indicators of SES including education, income, and home ownership; controlling for more aspects of SES than our study, yet still not able to fully account for the difference in prevalence of food insecurity between the indigenous and non-indigenous populations. The study employed a more comprehensive tool to measure food insecurity than we used in our study; the US Household Food Security Survey Module (HFSSM). They concluded however, that a shortcoming of the HFSSM was that it did not collect any information about the coping strategies of the food insecure and that it was therefore possible that differences in coping strategies may also have contributed to differences in the prevalence of food insecurity. Similarly, the VPHS only contained a single question pertaining to food insecurity and did not collect any information on coping strategies; such as, the use of food banks and the rationing of food. It is therefore possible that unmeasured differences in coping strategies may have contributed to the difference in the prevalence of food insecurity between Aboriginal and Torres Strait Islanders and their non-Aboriginal and Torres Strait Islander counterparts; which would warrant investigation.

### Strengths of the study

The VPHS is a population representative survey of the adult population of Victoria with a good response rate of 64.9% in 2008; comparable to that of the 2009 U.S National Health Interview Survey (65.4%).

The number of Aboriginal and Torres Strait Islanders recruited to the survey constituted 1% of the survey sample which was over-representative of the wider Victorian population (approximately 0.7%). Typically surveys tend to suffer from under-representation of the Aboriginal and Torres Strait Islander population due to reluctance to self-identify. One possible explanation for this is that the VPHS was not specifically aimed at the Victorian Aboriginal and Torres Strait Islander population and therefore respondents were not questioned about their Aboriginal or Torres Strait Islander status until the end of the survey, after the interviewer has established a rapport with the respondent, which may have been more conducive to identification.

### Limitations of the study

Although Aboriginal and Torres Strait Islanders were over-represented in the 2008 VPHS, the absolute sample size was only 339. However, the sample size had a power of 80.5% to detect an OR of 1.8, α = 0.05 (2-sided). Moreover, the RSEs for all prevalence estimates were less than 25% indicating a reasonable degree of statistical reliability.

We used a single question to measure food insecurity which fails to capture all facets of food insecurity. Therefore our estimate of the prevalence of food insecurity in Victoria is likely to be a very conservative estimate.

The data are self-reported and therefore factors such as smoking, excessive alcohol consumption and obesity may be under-reported. Moreover 18.3% of respondents refused or were unable to indicate their total annual household income; although this did not differ between Aboriginal and Torres Strait Islanders (14.8%; 9.6–22.1%) and non-Aboriginal and Torres Strait Islanders (18.3%; 17.6–19.0%). However, if this measurement error is randomly distributed across the study population (non-differential misclassification), it would be expected to drive the direction of the association between the outcome and primary exposure variable towards the null [21].

The data is cross-sectional and therefore causality and its direction cannot be inferred with such a study design.

While the response rate of the survey was 64.9%, a non-response analysis indicated a selection bias where males and people aged 18 to 34 years were under-represented [22]. This was corrected for by weighting the data by the sex, age and geographic distribution of the state as well as the probability of being selected. However, since the survey was conducted using computer-assisted telephone interviewing, a further selection bias was introduced by virtue of the fact that only people who could afford a landline telephone connection were included in the sample. Therefore there was an under-representation of very low SES adults. This means that we are likely to have under-estimated the true prevalence of food insecurity in Victoria. However, such a systematic bias does not invalidate our findings but rather suggests that the prevalence of food insecurity may be larger than we have been able to enumerate here.

## Conclusions

Food insecurity is a serious problem among Aboriginal and Torres Strait Islanders who reside in the Australian state of Victoria. Differences between Aboriginal and Torres Strait Islanders and their non-Aboriginal and Torres Strait Islander counterparts in age structure, SES (household income), prevalence of lifestyle risk factors and social support only partially explained the higher prevalence of food insecurity among Aboriginal and Torres Strait Islanders. Further research is needed to identify the reason(s) for the substantially higher prevalence of food insecurity among Aboriginal and Torres Strait Islanders in Victoria.

To our knowledge this is the first study of its kind to investigate the determinants of food insecurity among Aboriginal and Torres Strait Islander in the state of Victoria using a population representative approach.

Information on the health and well-being of Aboriginal and Torres Strait Islanders in Australia most often comes from national datasets and is often dominated by studies with a focus on remote rural communities. This is mainly due to pragmatic considerations relating to sample size; Aboriginal and Torres Strait Islanders only constitute 2.5% of the Australian population with 73% residing in three states: New South Wales, Queensland and Western Australia [23]. Our work therefore highlights the importance of being reminded that while a small population in size, the Aboriginal and Torres Strait Islander population is a culturally and linguistically diverse population; geographic location is an important determinant of that diversity.

Our work contributes to the overall understanding of food insecurity in Australia, which, to date, has largely been based on treating ethnicity as a risk factor for food insecurity. We confirm that income insufficiency is indeed a key determinant of food insecurity. However, we also show that income insufficiency is not in itself sufficient to explain food insecurity among Aboriginal and Torres Strait Islanders.

Future research on food insecurity among Aboriginal and Torres Strait Islanders should be directed at investigating all facets of food insecurity; including factors that influence the food supply, access to and utilisation of the food supply. Coping strategies for households at risk of food insecurity is another important area of focus; as is, understanding the role that institutional and other forms of racism (past and present) continues to play.

## Competing interests

The authors certify that there are no competing interests, financial or otherwise, regarding the material discussed in this manuscript.

## Authors’ contribution

AM designed the research; AM, Z.A, and MS conducted the research; AM analysed the data; AM, ZA, M.S, and JM wrote the paper; AM had primary responsibility for the final content. All authors read and approved the final manuscript.

## Pre-publication history

The pre-publication history for this paper can be accessed here:

http://www.biomedcentral.com/1471-2458/14/598/prepub
